# Huge Gastric Teratoma in an 8-Year Old Boy

**DOI:** 10.21699/ajcr.v7i5.495

**Published:** 2016-11-01

**Authors:** Rajpal S Sisodiya, Simmi K Ratan, Parveen k Man

**Affiliations:** Department of Pediatric Surgery, Maulana Azad Medical College and associated Lok Nayak Hospital, New Delhi-110002

**Keywords:** Gastric teratoma, Benign teratoma, Extra gonadal germ cell tumor

## Abstract

Gastric teratoma is very rare tumor and usually presents in early infancy. An 8-year-old boy presented with a huge mass in abdomen extending from epigastrium to the pelvis. Ultrasound and CT scan of abdomen revealed a huge mass with solid and cystic components and internal calcifications. The preoperative diagnosis was a teratoma but not specifically gastric one. At operation, it was found to be gastric teratoma. The mass was excised completely with part of the stomach wall. The histopathology confirmed it to be mature gastric teratoma. The rarity of the teratoma with delayed presentation prompted us to report the case.

## CASE REPORT

An 8-year-old male child presented with mass in abdomen and early satiety since last one year. General physical examination was unremarkable. Abdominal examination reveal huge mass occupying almost entire abdomen. Complete blood count, renal function test and alpha feto protein were within normal limits. Ultrasound (USG) revealed heterogeneous mass. Contrast enhanced CT abdomen revealed large multiloculated solid and cystic mass in right side of abdomen with calcification, and displacing bowel loops towards left side but organ of origin could not be commented upon. At laparotomy, there was huge solid cum cystic mass occupying whole of the abdomen. Cystic area was aspirated followed by excision of mass. Intraoperatively mass was arising from lesser curvature of stomach. Part of the stomach was removed and primary repair of stomach was done (Fig.1). Cut-open specimen showed presence of teeth and putaceous material. Biopsy was consistent with mature teratoma. Postoperative recovery was uneventful and at one year follow-up child is doing well.

**Figure:**
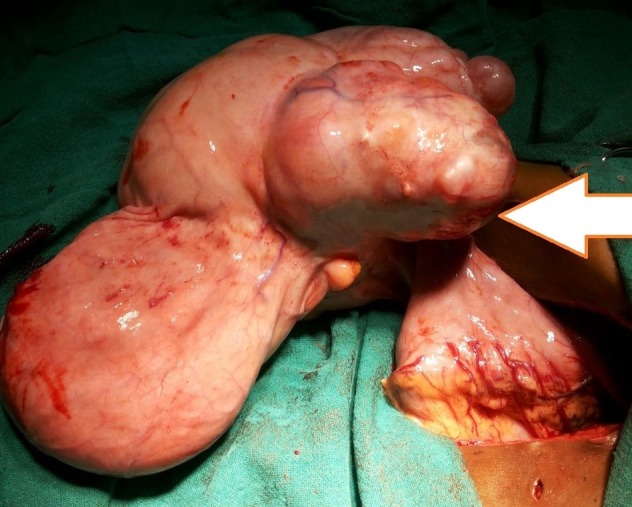
Figure 1:Gastric teratoma originating from stomach (Arrow).

## DISCUSSION

Sacrococcygeal teratoma is most common extra-gonadal teratoma in children whereas gastric teratoma is considered one of rarest types of it, accounting for less than 1% of cases. Extra-gonadal teratoma is more frequently seen in newborns, infants and toddlers; whereas, gonadal tumors occur more often in older children.[1,2] Most gastric teratomas are benign however malignant gastric teratomas are also reported in literature.[3] The diagnosis of teratoma can be made on radiological investigations. Our case presented late but outcome was satisfactory.

## Footnotes

**Source of Support:** Nil

**Conflict of Interest:** None declared

